# *N*-Demethylation and *N*-oxidation of imipramine in rat thoracic aortic endothelial cells

**DOI:** 10.1007/s11626-014-9739-0

**Published:** 2014-03-20

**Authors:** Yukari Ueda, Toshihiko Yaginuma, Eiko Sakurai, Eiichi Sakurai

**Affiliations:** 1 Department of Pharmaceutics, Faculty of Pharmaceutical Sciences, Tokushima Bunri University, 180 Yamashiro-cho, Tokushima, 770-8514 Japan; 2 Faculty of Pharmacy, Iwaki Meisei University, 5-5-1 Iino, Iwaki, Fukushima 970-8551 Japan

**Keywords:** Thoracic aortic endothelial cells (TAECs), Cytochrome P450 (CYP), Flavin-containing monooxygenase (FMO), Imipramine, *N*-Demethylation, *N*-Oxidation

## Abstract

The aim of this study was to examine whether cultured rat thoracic aortic endothelial cells (TAECs) have the ability to metabolize the tertiary amine, imipramine. In rat TAECs, imipramine was biotransformed into *N*-demethylate and *N*-oxide by cytochrome P450 (CYP) and flavin-containing monooxygenase (FMO), respectively. The intrinsic clearance (*V*_max_/*K*_m_) for the *N*-oxide formation was approximately five times as high as that for the *N*-demethylate formation, indicating that oxidation by CYP was much higher than that by FMO. Moreover, we suggest that CYP2C11 and CYP3A2 are key players in the metabolism to *N*-demethylate in rat TAECs using the respective anti-rat CYP antibodies (anti-CYP2C11 and anti-CYP3A2). The presence of CYP2C11 and CYP3A2 proteins was also confirmed in cultured rat TAECs using a polyclonal anti-CYP antibody and immunofluorescence microscopy. In contrast, the formation rate of *N*-oxide at pH 8.4 was higher than that at pH 7.4. Inhibition of *N*-oxide formation by methimazole was found to be the best model of competitive inhibition yielding an apparent *K*_i_ value of 0.80 μmol/L, demonstrating that *N*-oxidation was catalyzed by FMO in rat TAECs. These results suggest that rat TAEC enzymes can convert substrates of exogenous origin such as imipramine, indicating that TAECs have an important function for metabolic products, besides hepatic cells.

## Introduction

Endothelial cells have unique and efficient protective systems for controlling the passage of materials. The first is a permeability barrier, and the second appears to be a metabolic barrier formed by the enzymes in the endoplasmic reticulum of endothelial cells, which metabolize some of the permeable molecules recognized as substrates. Previous data indicate that isolated brain capillaries contain activities of enzymes involved in drug metabolism such as cytochrome P450s (CYPs), nicotinamide adenine dinucleotide phosphate (NADPH)–cytochrome P450 reductase, 1-naphthol glucuronyltransferase, and epoxide hydrolase (Ghersi-Egea et al. 1988). Similar to CYP, flavin-containing monooxygenases (FMOs) are microsomal enzymes that require NADPH and O_2_ and catalyze the oxidation of nucleophilic tertiary amines to *N*-oxides and secondary amines to hydroxylamines and oximes. The expression of FMO1, FMO2, and FMO5 proteins was confirmed in rat brain microvascular endothelial cells (BMECs) by western blotting analysis, suggesting that *N*-oxide of *d*-chlorpheniramine was formed in rat BMECs (Sakurai et al. 2013). Moreover, our previous data showed that for detoxification in rat lung microvascular endothelial cells (LMECs), nicotine was biotransformed into cotinine and nicotine *N*′-oxide by CYPs (CYP2C11 and CYP3A2) and FMO, respectively (Ochiai et al. 2006). Thus, it appears that in addition to hepatic cells, the microvascular endothelium is an important barrier for metabolic products, suggesting that this metabolic barrier may control the transfer of drug to the tissue. In contrast, because aortic endothelial cells construct the systemic circulation of the blood, the metabolic ability of drug in thoracic aortic endothelial cells (TAECs) may be different from that in BMECs and LMECs. But Borlak et al. (2003) already showed that the genes and proteins of major CYP monooxygenases, such as CYP2C8 and CYP2E1, are expressed in cultures of primary human coronary endothelial cells, and the endothelium has the ability to metabolize verapamil, a commonly and widely prescribed calcium antagonist. In the present study, we examined whether cultured rat TAECs have the ability to form *N*-demethylate by CYPs and *N*-oxide by FMO from the tertiary amine, imipramine, using enzyme inhibition or inactivation techniques, and investigated the significance of drug metabolic ability in rat TAECs.

## Materials and Methods

### Materials.

Imipramine *N*-oxide was synthesized, as described by Craig and Purushothaman (1970), and separated by high-performance liquid chromatography (HPLC), as described below. Imipramine, desipramine, glucose 6-phosphate dehydrogenase, NADP, and glucose 6-phosphate were obtained from Wako Pure Chemicals (Osaka, Japan). Gentamicin sulfate and amphotericin B were purchased from Sigma-Aldrich (St. Louis, MO). Dulbecco’s modified Eagle’s medium: nutrient mixture F-12 (D-MEM/F-12), heparin, piperazine-*N*′-(2-ethane-sulfonic acid) (HEPES), dispase, epidermal growth factor (EGF), fetal bovine serum (FBS), and donor horse serum (HS) were obtained from Gibco BRL, Life Technologies (Rockville, MD). Donkey serum was purchased from Abcam plc (Cambridge, UK). Dextran T-70 and Percoll were obtained from Pharmacia (Uppsala, Sweden). Collagenase P was purchased from Boehringer Mannheim (Mannheim, Germany). Anti-rat CYP2C11, CYP3A2, CYP1A1, and CYP2B1 goat sera and normal goat serum were purchased from Daiichi Pure Chemicals (Tokyo, Japan). Acetylated low-density lipoprotein labeled with a fluorescent probe, 1,1′-dioctadecyl-3,3,3′,3′ tetramethyl-indocarbocyamine perchlorate (DiI-Ac-LDL), was obtained from Biomedical Technologies (Stoughton, MA). All other chemicals were of reagent grade and commercially available.

### Isolation and culture of rat TAECs.

Three-wk-old male Wistar rats purchased from Japan SLC (Hamamatsu, Japan) were housed at a constant temperature (23 ± 1°C) and constant humidity (55 ± 5%), with automatically controlled lighting (0700–1900). Twenty rats were killed by decapitation, and the descending thoracic aorta 2–3 cm in length was removed. The vessel was rinsed several times in ice-cold phosphate-buffered saline (PBS), and the anterior end of the vessel was fastened to an 18.5-gauge hypodermic needle, which was attached to a 50-mL syringe filled with 0.07% collagenase P and 0.19% dispase. The vessel was then perfused with collagenase P and dispase at a flow rate of 1 mL/min at 37°C, and fractions of 10 mL were collected. Collected cells were filtered through 100-μm mesh. The rat TAECs were collected by centrifugation at 600×*g* for 10 min and resuspended in M199. The cell suspensions were seeded onto collagen-coated 75-cm^2^ tissue culture flasks (Iwaki Glass, Funabashi, Japan). Cells were allowed to attach and grow to monolayers at 37°C in a humidified atmosphere of 5% CO_2_/95% air. The culture medium (D-MEM/F-12 containing 14 mM of sodium bicarbonate, 20 ng/mL of EGF, 50 μg/mL of gentamycin–amphotericin B solution, 10 U/mL of heparin, 5% FBS, and 5% HS) was changed every 3 d. Subculture was performed when the cells reached confluence, after approximately 6–7 d. Cells were trypsinized at a ratio of 1:3 after reaching confluence using 0.025% trypsin in HBSS containing 0.02% EDTA. Secondary subcultured cells (5 × 10^5^ cells/cm^2^) were grown on collagen-coated 225-cm^2^ tissue culture flasks. All metabolism experiments were only performed on TAECs that had undergone two passages, after cells reached confluence in approximately 4–5 d.

### Immunofluorescence analysis.

Rat TAECs grown on collagen-coated culture slides (FALCON, Bedford, MA) were fixed in 3% paraformaldehyde in PBS at room temperature for 10 min. The cells were permeabilized with 0.5% Triton X-100 for 5 min and rinsed with PBS. The cells were incubated in 5% donkey serum in PBS for 10 min and then in polyclonal goat anti-rat CYP2C11 antibody (1:500) and rabbit anti-rat CYP3A2 (1:500) in the same solution for 60 min. The control data were replaced by donkey polyclonal anti-goat IgG antibody and anti-rabbit IgG antibody (Invitrogen, Carlsbad, CA). After rinsing, the cells were incubated with donkey anti-goat IgG-fluorescein isothiocyanate (FITC) conjugate (1:1,000) and donkey anti-rabbit IgG-FITC conjugate (1:1,000) for 60 min and mounted for observation and photography. Culture slides, which received only the secondary antibody, served as negative controls.

### Enzyme assay.

For enzyme kinetic studies, cultured rat TAECs were homogenized in 0.1 mol/L phosphate buffer (pH 7.4 and pH 8.4). Incubation vessels contained rat TAECs (0.25 mg protein/mL), MgCl_2_ (25 mmol/L), glucose 6-phosphate (6.7 mmol/L), nicotine amide (2.5 mmol/L), and glucose 6-phosphate dehydrogenase (1 U/mL) in a total volume of 2 mL. Imipramine dissolved in 0.1 mol/L phosphate buffer (pH 7.4 and pH 8.4) was the substrate at a final concentration in the range of 5.0–100.0 μmol/L. After addition of NADP (0.5 mmol/L in 0.1 mol/L phosphate buffer), the mixtures were incubated for 2 min at 37°C in a shaking water bath. At the end of the incubation, 1 mL of 5.0 mol/L NaOH and 7 mL of ethyl acetate were added to stop the reaction. The mixture was vigorously shaken for 10 min and centrifuged for 10 min at 800×*g*. The organic layer (5 mL) was evaporated to dryness under N_2_. The residues were redissolved in 200 μL of the mobile phase of HPLC as described below, and 20 μL was injected onto an HPLC column. Protein concentrations were assayed using the method of Markwell et al. (1978).

### Inhibition study.

To characterize CYP isoforms in rat TAECs, five polyclonal antibodies were used for the inhibition study: anti-CYP1A1, anti-CYP2B1 that cross-reacts with CYP2B2, anti-CYP2C11 that cross-reacts with CYP2B1 and CYP2B2, and CYP3A2. Each anti-CYP serum (10 μL) was incubated with 25 μL of rat TAEC homogenate (500 μg protein) for 30 min at 37°C. The same volume of normal goat serum was incubated with the rat TAECs to determine a nonspecific reaction. After this period, 155 μL of the reaction mixture containing 25 μmol/L imipramine was added to the reaction vessels. All other incubation conditions were as described. Methimazole was also used for the inhibition study of FMO. Inhibition kinetics for methimazole were determined in rat TAEC homogenates using the standard assay procedure with five different imipramine concentrations and methimazole (1 μmol/L). Data were analyzed by nonlinear regression analysis to allow determination of the type of inhibition and inhibitory constants.

### HPLC condition.

HPLC was performed using a Shimadzu (Kyoto, Japan) LC-6A apparatus equipped with an ultraviolet (UV) detector (Shimadzu SPD-10AVP, Kyoto, Japan) and LiChrospher Si60 column (250 × 4.6 mm i.d., 5 μm particle size; Kanto Chemical Co., INC, Tokyo, Japan). Material was eluted with acetonitrile/methanol/28% ammonia water (73:25:2, *v*/*v*) at a flow rate of 1 mL/min at 30°C, and the absorption at 228 nm was measured. The peaks of imipramine *N*-demethylate (desipramine) and synthesized imipramine *N*-oxide were symmetrical and clearly separated from other peaks. The calibration curve for metabolites was linear over the concentration range 50 μg/mL, and the lower limit for quantitation was 0.1 μg/mL.

### Data analysis.

The formation of metabolites from imipramine was calculated as nmol formed/min/mg protein. Kinetic data were fit to the Michaelis–Menten equation for a one-enzyme or two-enzyme system using the nonlinear least-squares regression analysis program MULTI, and apparent *K*_m_ and *V*_max_ values were estimated. Values were presented as mean ± standard error of the mean for *n* experiments. Comparisons of data among groups were performed using an analysis of variance and Dunnett’s post hoc multiple comparisons test. Differences were considered to be significant at *P* < 0.05 (two-tailed).

## Results

### The formation rate of imipramine *N*-demethylate and *N*-oxide in rat TAECs.

**Figure 1.**
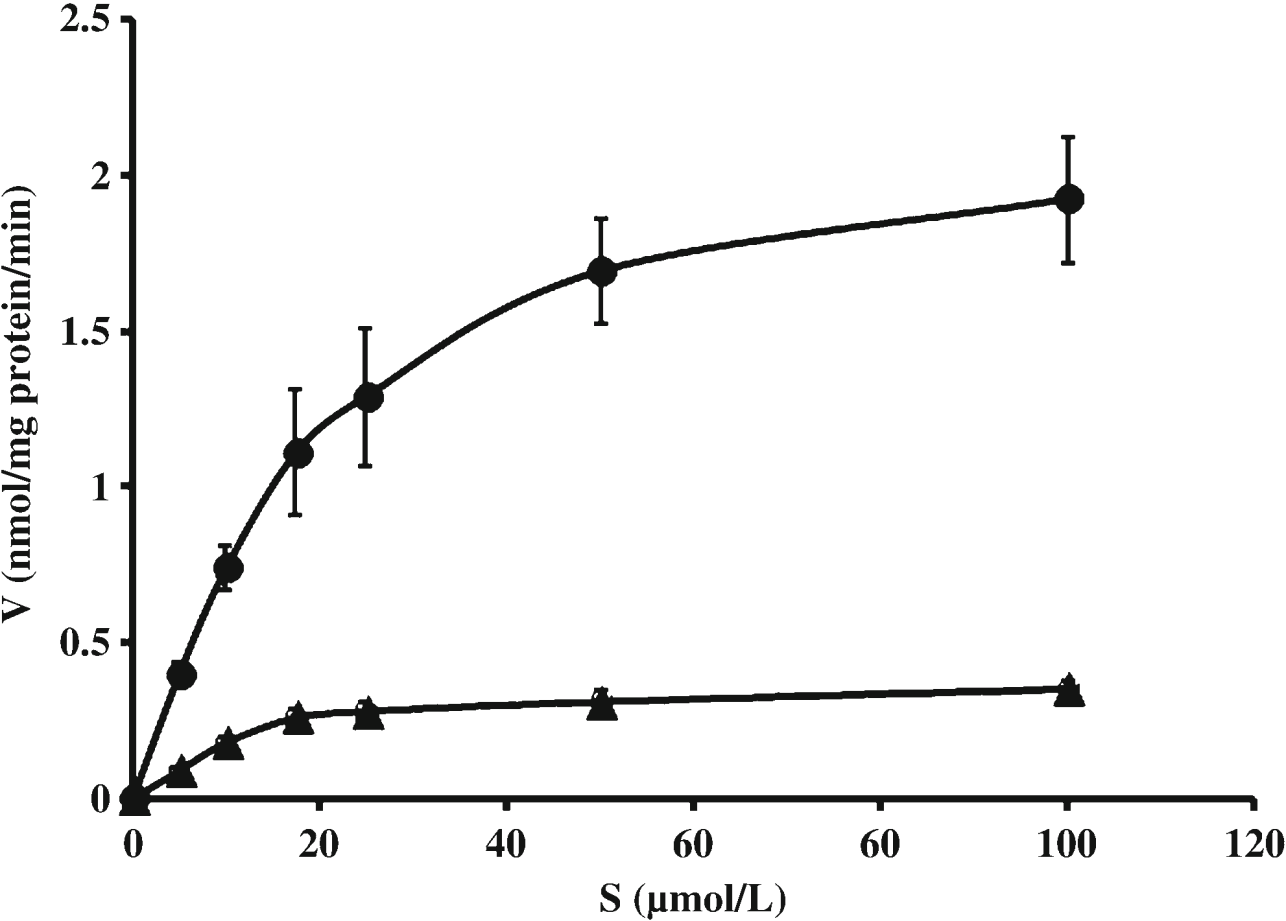
Michaelis–Menten plots for formation of desipramine (*closed triangle*) and imipramine *N*-oxide (*closed circle*) from imipramine incubated with homogenized rat TAECs. Imipramine metabolism was determined at 37°C for 2 min at concentrations between 5.0 and 100 μmol/L. Each *point* represents the mean ± SE of five experiments. *V* metabolite formation rate (nmol/mg protein/min), *S* imipramine concentration (μmol/L).

**Figure 2.**
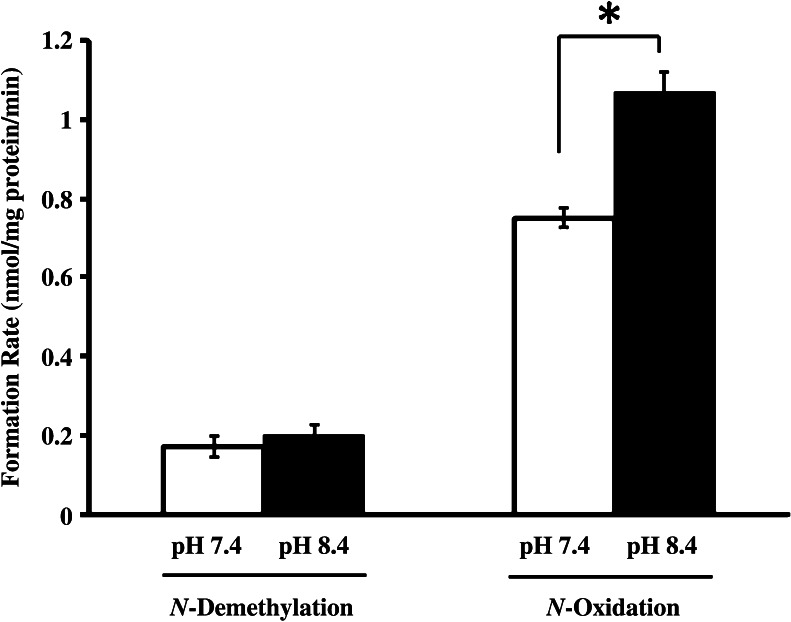
Effect of pH on metabolism of imipramine by rat TAECs homogenates. Imipramine metabolism was determined with 0.1 mol/L phosphate buffer at pH 7.4 or pH 8.4. Each value is expressed as the mean ± SE of five experiments. *P < 0.05 indicates a statistically significant difference between pH 7.4 and pH 8.4.

The formation of imipramine *N*-demethylate and *N*-oxide from imipramine (5.0–100 μmol/L) incubated with cultured rat TAECs were fit to Michaelis–Menten plots, respectively (Fig. 1). Consequently, the intrinsic clearance value (*V*_max_/*K*_m_) for *N*-oxidation was higher than that for *N*-demethylation at pH 7.4 (Table 1). Moreover, although there was no significant difference in *N*-demethylation activity at pH 7.4 or pH 8.4, the formation of imipramine *N*-oxide at pH 8.4 was significantly higher compared with that at pH 7.4 (Fig. 2).

**Table 1.** Kinetic parameters for *N*-demethylate and *N*-oxide formation from imipramine in rat TAECs

	*K*_m_ (μmol/L)	*V*_max_ (nmol/mg protein/min)	*V*_max/_ *K*_m_ (mL/mg protein/min)
*N*-Demethylation	39.9 ± 5.3	0.86 ± 0.42	0.022 ± 0.003
*N*-Oxide	20.0 ± 4.5	2.27 ± 0.27	0.114 ± 0.014

Each value is expressed as the mean ± standard error of five experiments

### The inhibitory effect of anti-CYP antibodies on the *N*-demethylation activity in rat TAECs.

**Figure 3.**
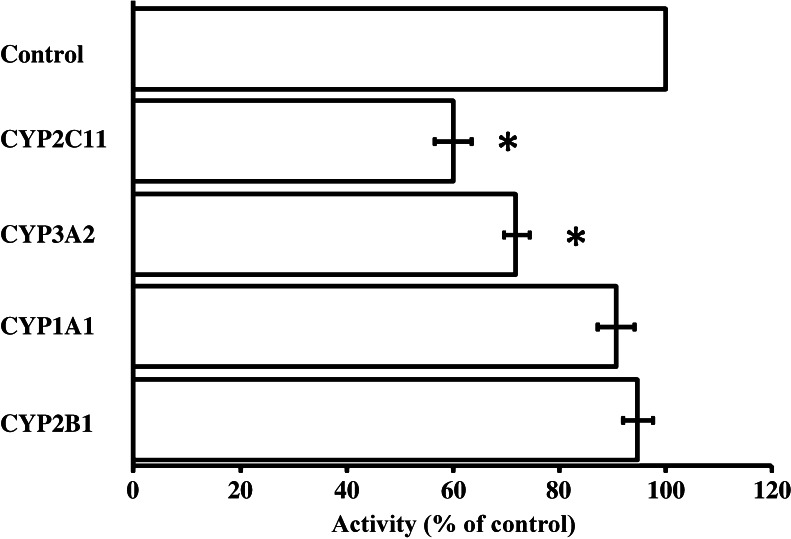
The inhibitory effect of anti-CYP antibodies on the *N*-demethylation activity in rat TAECs. The rat TAEC homogenate proteins (500 μg) were combined with 10 μL each of various anti-rat CYP sera and normal goat or rabbit serum and incubated at 37°C for 30 min before adding the reaction mixture containing 25 μmol/L imipramine. Values for the antibody-treated group are expressed as a percentage of activity of the control group.

Addition of 25 μL anti-CYP2C11 and anti-CYP3A2 antibodies to the reaction mixture containing 500 μg of rat TAECs protein inhibited the formation of *N*-demethylate from imipramine to 60.0% and 71.8% of the control values for imipramine, respectively. But anti-CYP1A1 and anti-CYP2B1 had no inhibitory effects on the rate of *N*-demethylation of imipramine (Fig. 3).

### Expression of CYP2C11 and CYP3A2 proteins in rat TAECs.

**Figure 4.**
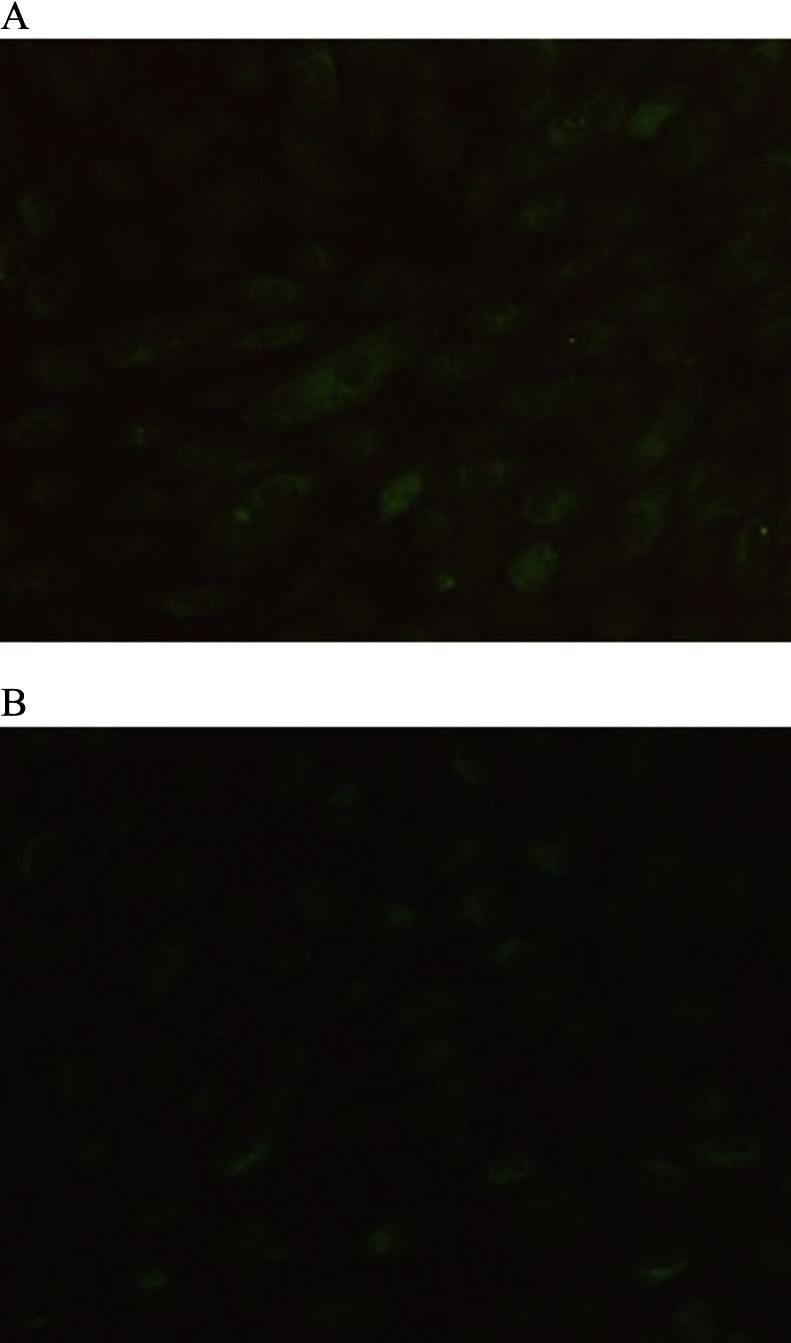
Indirect immunofluorescent histochemistry of rat TAECs using the polyclonal anti-CYP 2C11 (*A*) and anti-CYP 3A2 (*B*) antibodies.

The presence of CYP2C11 and CYP3A2 was confirmed by fluorescence microscopy using the polyclonal anti-CYP antibodies (Fig. 4A, B). The secondary antibody control of the staining was negative (data not shown).

### The inhibitory effect of methimazole on imipramine *N*-oxide formation in rat TAECs.

**Figure 5.**
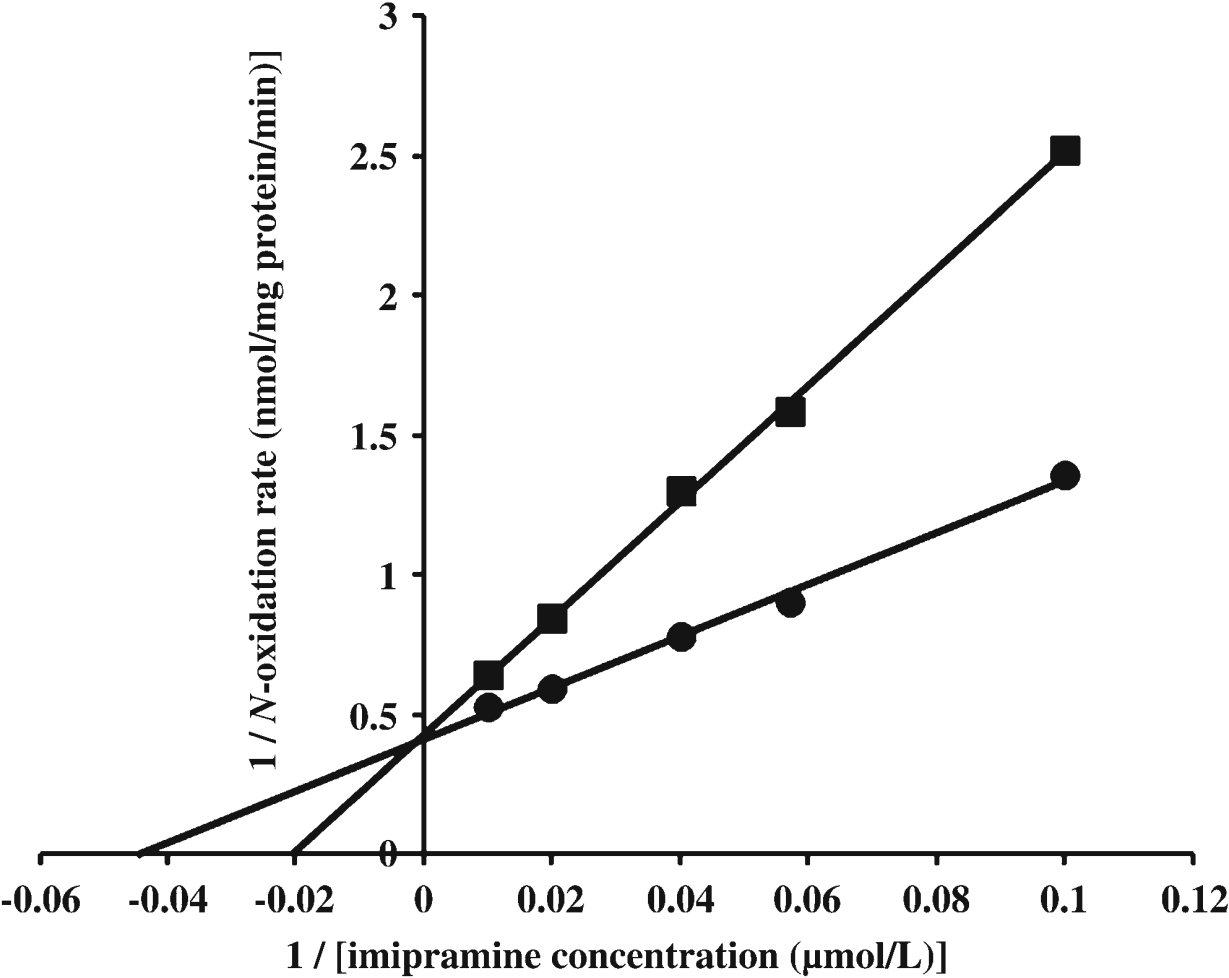
Inhibition of imipramine *N*-oxidation by methimazole in rat TAECs. Lineweaver–Burk plots of the reciprocal of the initial velocity of imipramine *N*-oxidation against the reciprocal of the imipramine concentration in the presence of methimazole. Each *line* is the best fit through the mean of FMO activities for three data points. *Closed circle* no drug added; *closed square* mechimazole (1 μmol/L).

Methimazole, an enzyme inhibitor of FMO activity, competitively inhibited FMO activity, and the *K*_i_ value was estimated to be 0.80 μmol/L using Lineweaver–Burk plots (Fig. 5).

## Discussion

The drug appears to be absorbed from the site of administration into the blood and is then distributed to the tissues. The transfer of drug to the tissues is important for local drug response, whereas the accumulation of the drug in the tissues has toxic effects. Therefore, transfer of drug to the tissues may be limited in endothelial cells. Because drug in the blood is always exposed to oxygen, the importance of drug biotransformation in endothelial cells is recognized. Several investigators reported that CYP-dependent drug oxidation activity in endothelial cells and the induction of endothelial CYP monooxygenases can be achieved with various chemical agents (Farin et al. 1994; Graier et al. 1995; Stegemann et al. 1995; Adeagbo 1997). We also reported that similar to CYP, FMOs that oxidize the nucleophilic nitrogen, sulfur, and phosphorus heteroatom of a variety of xenobiotics exist in cultured rat brain and LMECs (Ochiai et al. 2006; Sakurai et al. 2013).

In centrally acting drugs, there are many compounds that have the chemical structure of a tertiary amine with two methyl groups attached to the basic nitrogen atom. Some tricyclic antidepressants, such as imipramine and amitriptyline, also have the structure of a tertiary amine and have pronounced effects in the central nervous system. Tertiary amines are metabolized in the liver by two main routes, namely *N*-demethylation by CYP, whereby tertiary amines are converted to secondary amines, and *N*-oxidation by FMO. This metabolism may occur in TAECs to protect the brain and other tissues. In this study, we have quantified the activities of two key imipramine metabolizing enzymes, CYP and FMO, and have also characterized isoforms in cultured rat TAECs.

Our results indicate that imipramine *N*-oxide is predominantly formed from imipramine, whereas imipramine *N*-demethylate is a relatively minor metabolite in rat TAECs. The metabolic route of imipramine involves primarily *N*-demethylation to desipramine and aromatic hydroxylation to 2-hydroxy-imipramine, which are catalyzed by hepatic microsomal P450 in experimental animals and humans. Because human and rat liver microsomes are a small extent in imipramine *N*-oxide formation, the metabolic profile obtained by rat TAECs is different from that obtained by rat liver.

Thum and Borlak (2000) showed that rat aortic endothelial cells express several genes (CYP1A1, CYP2B1/2, CYP2C11, and CYP2E1) that code for drug-metabolizing enzymes. They also found CYP1A1, CYP2A6/7, CYP2A13, CYP2B6/7, CYP2C8, CYP2E1, CYP2J2, and cyclophilin (housekeeping gene) to be expressed in cultures of human coronary arterial endothelial cells, but transcript levels of other CYPs were below the limit of detection (Borlak et al. 2003). In this report, characterization of the CYP isoenzymes involved in *N*-demethylation of imipramine was investigated using anti-rat CYP antibodies (anti-CYP2C11, anti-CYP3A2, anti-CYP1A1, and anti-CYP2B1). As shown in Fig. 3, the immunoinhibition study suggests that CYP2C11 and CYP3A2 are the major CYP isoenzymes involved in the *N*-demethylation of imipramine in cultured rat TAECs. CYP2B1 and CYP2B2 are also present in the hepatic microsomes of untreated male rats at low levels (Guengerich et al. 1982; Christou et al. 1987; Imaoka et al. 1989; Yamazoe et al. 1987). In contrast, the two major CYP isoenzymes, CYP1A1 and CYP1A2, are potently induced by 3-methylcholanthrene (Degawa et al. 1988; Juedes and Kupfer 1990). However, anti-CYP2B1 and anti-CYP1A1 did not clearly inhibit *N*-demethylation of imipramine in cultured rat TAECs. Moreover, as shown in Fig. 4, we also confirmed the presence of CYP2C11 and CYP3A2 proteins in cultured rat TAECs using a polyclonal anti-CYP antibody and immunofluorescence microscopy, suggesting that characterization of the CYP isoenzymes involved in oxidation in rat TAECs is similar to that in rat LMECs (Ochiai et al. 2006). On the other hand, because imipramine and desipramine are oxidized at the 2-position by CYP2D, further study on the relation between the expression of CYP2D protein and 2-hydroxy-imipramine formation is necessary in cultured rat TAECs.

In general, FMO is relatively thermolabile and has a higher optimal pH in the reaction compared with reactions mediated by CYP (Ziegler 1988). Therefore, the condition of these enzymes in some reactions can be altered by the experimental conditions such as pH. In this study, the formation rate of *N*-oxide at pH 8.4 was higher than that at pH 7.4 in cultured rat TAECs, suggesting that FMO is the enzyme responsible for the formation of imipramine *N*-oxide. However, there was no significant difference in the formation of *N*-demethylate at pH 7.4 or pH 8.4. Moreover, to determine the contribution of the FMO enzyme with the formation of imipramine *N*-oxide, we examined the effect of methimazole on this activity. Methimazole is a well-known inhibitor of FMO (Ziegler 1988). As shown in Fig. 5, the formation of imipramine *N*-oxide was competitively inhibited by methimazole in a dose-dependent manner, demonstrating the presence of the FMO enzyme in rat TAECs. Till date, five isoforms of FMO (FMO1–FMO5) have been identified in humans (Burnett et al. 1994; Hines et al. 1994). Other mammals also express different FMOs in a species- and tissue-specific manner (Ziegler 2002). Therefore, further experiments on characterization of FMO isoforms will be necessary in cultured rat TAECs.

## Conclusions

Rat TAEC enzymes can convert substrates of exogenous origin, such as imipramine for detoxification, indicating that TAECs have an important function for metabolic products, in addition to being a permeability barrier to the passage of materials.
